# Resistance to the Antiproliferative In Vitro Effect of PI3K-Akt-mTOR Inhibition in Primary Human Acute Myeloid Leukemia Cells Is Associated with Altered Cell Metabolism

**DOI:** 10.3390/ijms19020382

**Published:** 2018-01-27

**Authors:** Ina Nepstad, Håkon Reikvam, Annette K. Brenner, Øystein Bruserud, Kimberley J. Hatfield

**Affiliations:** 1 Department of Clinical Science, University of Bergen, 5021Bergen, Norway; ina.nepstad@uib.no (I.N.); annette.brenner@uib.no (A.K.B.); Oystein.Bruserud@uib.no (Ø.B.); 2 Department of Medicine, Haukeland University Hospital, 5021 Bergen, Norway; Hakon.Reikvam@uib.no; 3 Department of Immunology and Transfusion Medicine, Haukeland University Hospital, 5021 Bergen, Norway

**Keywords:** acute myeloid leukemia, metabolism, mTOR, PI3K, phosphorylation

## Abstract

Constitutive signaling through the phosphatidylinositol-3-kinase-Akt-mechanistic target of rapamycin (PI3K-Akt-mTOR) pathway is present in acute myeloid leukemia (AML) cells. However, AML is a heterogeneous disease, and we therefore investigated possible associations between cellular metabolism and sensitivity to PI3K-Akt-mTOR pathway inhibitors. We performed non-targeted metabolite profiling to compare the metabolome differences of primary human AML cells derived from patients susceptible or resistant to the in vitro antiproliferative effects of mTOR and PI3K inhibitors. In addition, the phosphorylation status of 18 proteins involved in PI3K-Akt-mTOR signaling and the effect of the cyclooxygenase inhibitor indomethacin on their phosphorylation status was investigated by flow cytometry. Strong antiproliferative effects by inhibitors were observed only for a subset of patients. We compared the metabolite profiles for responders and non-responders towards PI3K-mTOR inhibitors, and 627 metabolites could be detected. Of these metabolites, 128 were annotated and 15 of the annotated metabolites differed significantly between responders and non-responders, including metabolites involved in energy, amino acid, and lipid metabolism. To conclude, leukemia cells that are susceptible or resistant to PI3K-Akt-mTOR inhibitors differ in energy, amino acid, and arachidonic acid metabolism, and modulation of arachidonic acid metabolism alters the activation of mTOR and its downstream mediators.

## 1. Introduction

Acute myeloid leukemia (AML) is a heterogeneous malignancy characterized by proliferating myeloblasts in the bone marrow [1,2]. Abnormal constitutive signaling through intracellular pathways is often observed in AML, including the phosphatidylinositol-3-kinase-Akt-mechanistic/mammalian target of rapamycin (PI3K-Akt-mTOR) pathway that seems to be important both in normal and leukemic hematopoiesis [3,4,5]. This pathway is important for regulation of proliferation, apoptosis, differentiation, and metabolism [6,7]. 

Constitutive signaling through the PI3K-Akt-mTOR pathway is found in 50–80% of AML patients and correlates with poor prognosis [4,8]. This abnormal signaling may be initiated by various mechanisms, e.g., oncogenes or mutated receptor tyrosine kinases, cell adhesion molecules, G-protein-coupled receptors, or other cytokine or hormonal receptors. 

When signaling is initiated in response to extracellular stimuli, scaffolding proteins are recruited and bind to the regulatory subunit of PI3K. Sequentially, PI3K phosphorylates phosphatidylinositol (4,5)-bisphosphate (PIP2) to generate phosphatidylinositol (3,4,5)-trisphosphate (PIP3), which facilitates recruitment and binding of proteins containing pleckstrin–homology domains, including Akt and its upstream activator 3’phosphoinositide-dependent kinase 1 (PDK1) [9]. PDK1 phosphorylates Akt at T308, leading to its partial activation. However, a subsequent phosphorylation at S473 is required for full enzymatic activation of Akt [9,10], and this can be achieved by mTOR complex 2 (mTORC2) or DNA-dependent protein kinase (DNA-PK) [10,11]. The mTOR kinase is part of two complexes, mTORC1 and mTORC2 with different biochemical structures and substrate specificity. The interactions between Akt and mTORC1/2 are complex. Akt phosphorylates the inhibitor of mTORC1 and proline-rich Akt-substrate-40 (PRAS40), preventing the suppression of mTORC1 signaling. Additionally, an Akt-driven inactivation of tuberous sclerosis complex (TSC) 1/2, leads to activation of mTORC1 through Ras homolog enriched in brain (RHEB). mTORC1 is an important regulator of cellular metabolism and protein synthesis through phosphorylation and activation of both the S6 ribosomal protein kinase (S6PK) and the repressor of messenger RNA (mRNA) translation initiation factor 4E-binding protein 1 (4EBP1) [6].

The PI3K-Akt-mTOR signaling pathway is one of the most frequently dysregulated pathways in human malignancies, including AML [12,13]. Though PI3K-Akt-mTOR is rarely mutated in AML, these patients harbor several mutations that may activate the pathway [12,14] and, hence, contribute to chemoresistance [4,15]. Based on extensive experimental studies, this pathway has been regarded as a possible therapeutic target in human AML [3,16]. However, despite this evidence, the initial clinical studies suggest that the tested mTOR inhibitors have only a modest antileukemic effect [16]. However, it should be emphasized that previous experimental studies also suggest that therapeutic targeting of the PI3K-Akt-mTOR pathway will be effective only for a subset of patients [17], and pathway inhibition may be more effective when using combined treatment strategies [18].

As discussed in a recent review, previous studies of resistance towards PI3K-Akt-mTOR inhibitors have focused on the possible hyperactivation of upstream mediators through feedback loops (e.g., PI3K and Akt) and compensatory activation of other pathways [16]. However, this cannot be the only explanation because chemoresistance is also seen for inhibitors upstream to Akt [17]. Our present study is to the best of our knowledge the first to suggest that metabolic alterations are a part of the therapy-resistant AML cell phenotype, and the metabolic differences seem to involve pathways that are involved in cellular energy and amino acid metabolism.

## 2. Results

### 2.1. Selection of Patients for the Metabolomics Comparison of Primary Human Acute Myeloid Leukemia (AML) Cells

We investigated the antiproliferative effect of four PI3K-Akt-mTOR inhibitors on primary human AML cell proliferation in the presence of exogenous cytokines [17]. The antiproliferative effects of the inhibitors differed considerably between the 56 patient cell samples studied, and for a subset of patients, even increased proliferation was seen in the presence of pathway inhibitors. In contrast, these previous studies showed that pathway inhibitors had only minor effects on the spontaneous stress-induced in vitro apoptosis that occurs during culture of primary human AML cells [19], and only minor differences could then be detected between different patients [17]. Patient subsets could thus be identified based solely on differences in our proliferation assay, i.e., these cells survived for 6 days in the presence of the pathway inhibitors and could still proliferate and incorporate ^3^H-thymidine during the period from day 6 to day 7 of the in vitro culture. This means that our subclassification was based on the pharmacological effects on a cell subset within the hierarchically organized AML cell population that were able to both survive and still be able to proliferate. 

**Figure 1:**
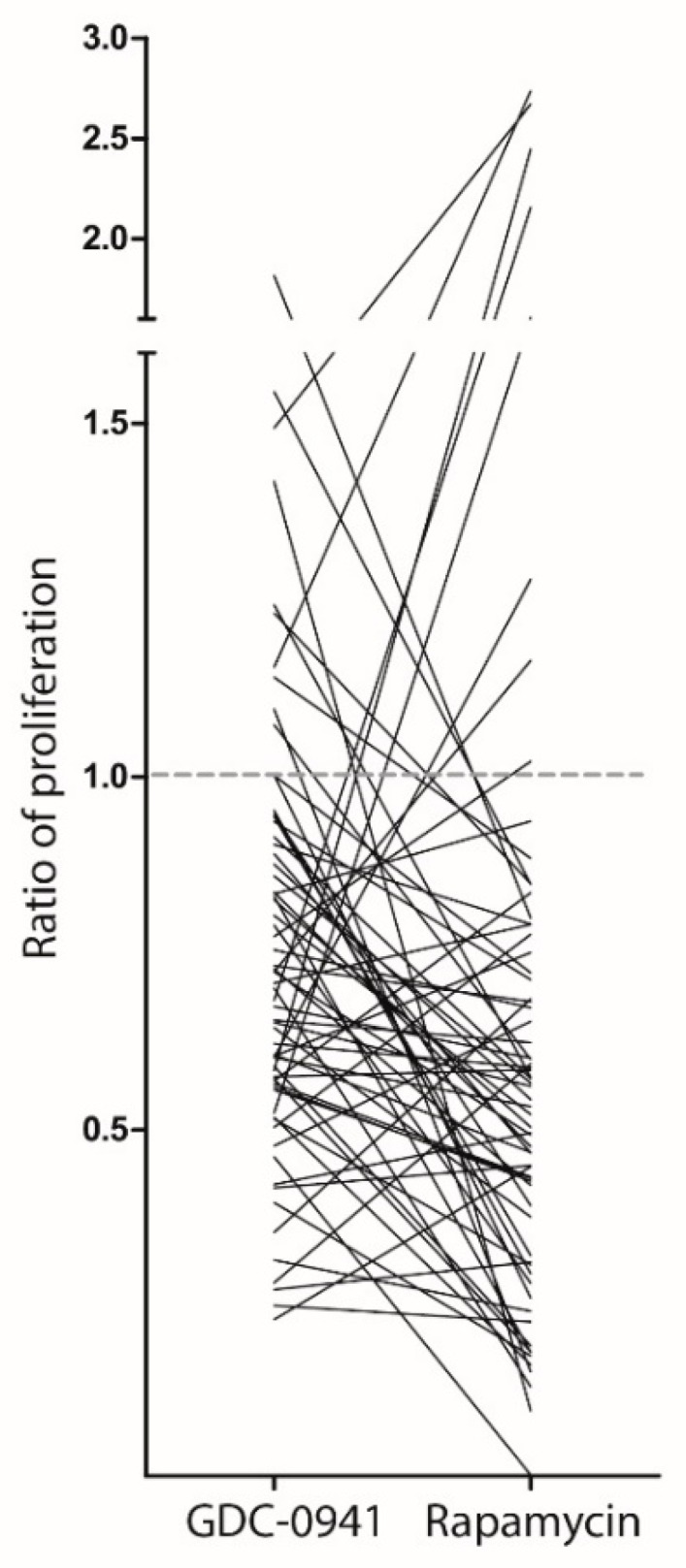
The effect of phosphatidylinositol-3-kinase-mechanistic target of rapamycin (PI3K-mTOR) inhibitors on cytokine-dependent in vitro acute myeloid leukemia (AML) cell proliferation. Leukemic cell proliferation was assayed as ^3^H-thymidine incorporation after six days of culture. We compared the proliferation of primary human AML cells cultured in the presence of the PI3K-inhibitor GDC-0941 and the mTOR-inhibitor rapamycin. The results are presented as the ratio of proliferation, i.e., nuclear incorporation of ^3^H-thymidine in drug-exposed cells relative to the incorporation in corresponding drug-free control cultures. The patient cohort included 76 patients, but detectable proliferation was only seen for the 68 AML patients whose results are presented in the figure. Each line represents the results for one patient. The dashed line indicates a ratio of 1.0, i.e., no change in proliferation.

We also investigated the effects of pathway inhibitors on the in vitro proliferation of primary human AML cells for a second and larger cohort including 76 additional consecutive patients; in these experiments, we only examined the effects of rapamycin and GDC-0941. The overall results are presented in Figure 1. The studies of this second cohort confirmed that the antiproliferative effects of PI3K-Akt-mTOR pathway inhibition varied among individual patients, and a variation of the effect between the two drugs was observed. We also investigated the susceptibility to stress-induced or spontaneous in vitro apoptosis for these 76 patients, but we could not observe any correlation between this susceptibility to apoptosis and the antiproliferative effects of the two pathway inhibitors. Taken together, our results from the two patient cohorts showed that neither the general regulation of apoptosis, as reflected in the degree of spontaneous in vitro apoptosis, nor the viability of the AML cell population after in vitro exposure to pathway inhibitors showed any significant association with the variation in antiproliferative effects of pathway inhibitors that was detected in our proliferation assay.

The data presented in Figure 1 clearly illustrate that pathway inhibitors can increase AML cell proliferation for a subset of patients, whereas for other patients, a strong inhibition corresponding to more than 50% inhibition could be detected for different mediators. For further analysis of the possible association between metabolic characteristics and the antiproliferative effects of pathway inhibitors on primary human AML cells, we compared two contrasting groups of selected patients based on the studies of the two patient cohorts. We then selected 15 patient samples with significantly decreased proliferation after inhibition with both rapamycin and GDC-0941; these samples are referred to as responders to the treatment. The other group included 15 patient samples showing no significant alteration of proliferation (corresponding to <10% inhibition) or even growth enhancement in the presence of pathway inhibitors. These are referred to as non-responders to treatment.

### 2.2. Patient Samples with Different Drug Sensitivity towards PI3K-mTOR Inhibitors Also Differ in Energy, Amino Acid and Arachidonic Acid Metabolism

**Figure 2:**
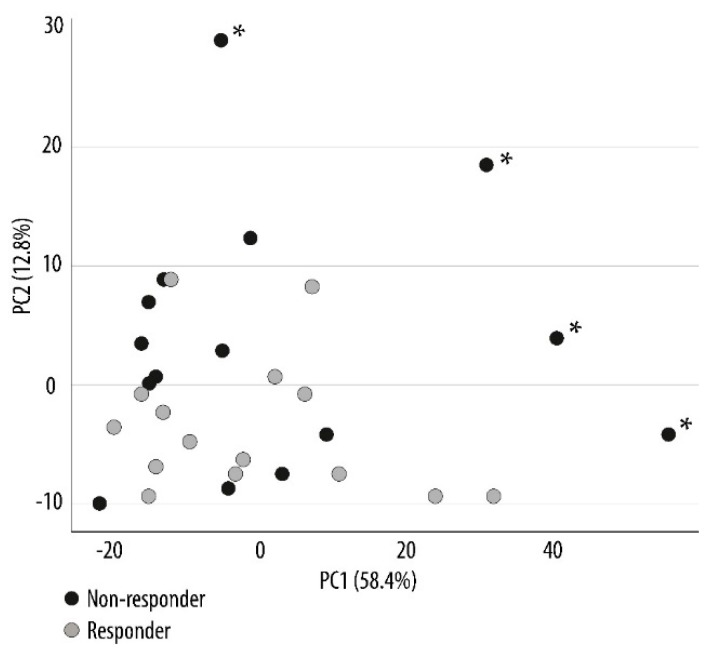
Principal component analysis (PCA) comparing the metabolic profiles of responders and non-responders to PI3K-mTOR inhibitors. The analysis was performed to generate an overview of the metabolic variance among the entire set of samples. Prior to this analysis, primary AML cells from 30 patients were separated into two contrasting groups based on their susceptibility to the in vitro antiproliferative effect of pathway inhibitors. The metabolic profiles for the primary AML cells derived from patients being susceptible (15 responders; grey circle) or resistant (15 non-responders; black circle) to PI3K-mTOR inhibitors were compared. The PCA depicts 71.2% (58.4 + 12.8% as indicated at the *X* and *Y* axis) of all variances in the data set. A separation of four non-responders (indicated by the asterisks *) from the rest of the sample was seen. Each circle represents the results for one patient.

Previous studies suggest that metabolic regulation of chronic myeloid leukemia cells is important for their susceptibility towards targeted therapy with kinase inhibitors [20]. We compared the metabolic profiles of the two contrasting patient groups that were sensitive and insensitive to PI3K-Akt-mTOR inhibition in vitro. As described above, these groups were selected based on their susceptibility to the antiproliferative effect of PI3K and mTOR inhibitors [17]. The metabolic analysis of the AML cells detected a total of 627 metabolites, and 128 of these metabolites were annotated. A principal component analysis was performed to illustrate the variance between the different sample groups. The responders and non-responders showed a great overlap in the plot, though four of the non-responders clustered separately from the rest (Figure 2). Thus, responders and non-responders could not be separated by an analysis of the overall metabolic profile, and non-responder patients seem to be heterogeneous with regard to their global metabolite profiles even though they show a similar resistance to PI3K-Akt-mTOR pathway inhibition.

Of the 627 detected metabolites, 23 metabolites differed significantly between the two contrasting groups of responders and non-responders, and among these, 15 were annotated (Table 1). These significantly altered metabolites are involved in energy (citric acid, isocitric acid, glutamine), amino acid (proline, glutamine, taurine), and lipid metabolism (two phosphatidylinositols (PI), the arachidonic acid metabolites 4,7,10,13-eicosatetraenoic acid, and 4,7,10,13,16-docosapentaenoic acid).

We did a metabolic pathway mapping based on the identified metabolites for further characterization of differences between responders and non-responders based on the 80 top-ranked metabolites. Purine metabolism (including the annotated metabolites glutamine, glutamic acid, glycine, and hypoxanthine) and Warburg effect (i.e., energy metabolism; including the annotated metabolites NAD, glutamic acid, glutamine citric acid, and isocitric acid) were then the two highest-ranked terms. This metabolic pathway mapping analysis further supports our conclusion that responders and non-responders to PI3K-mTOR inhibitors show metabolic differences.

### 2.3. Responders and Non-Responders to PI3K-Akt-mTOR Inhibition Could Be Identified Based on Metabolic Differences

**Figure 3:**
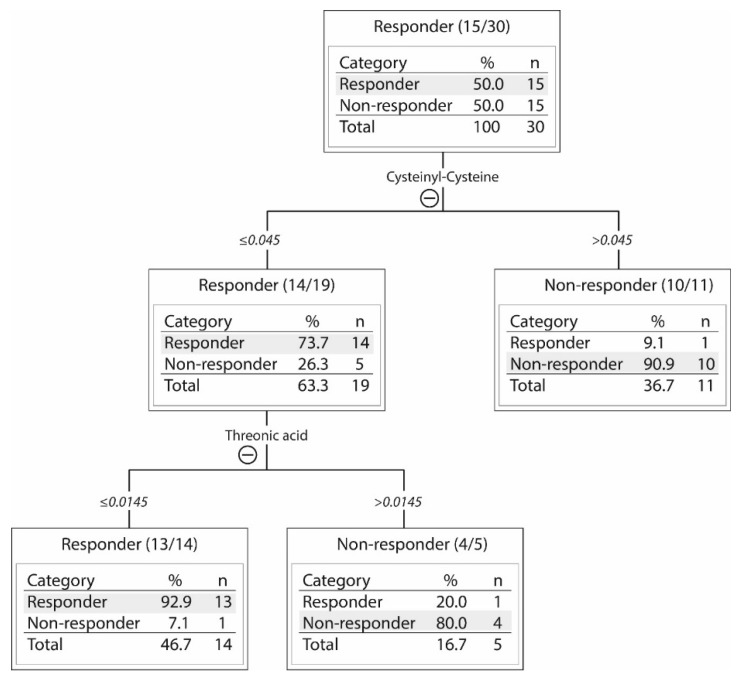
A decision tree analysis of the metabolic differences between 15 responders and 15 non-responders to PI3K-Akt-mTOR inhibition. The levels of two metabolites, cysteinyl-cysteine and threonic acid, allowed for discrimination between responders and non-responders. The 30 patients (see the upper box) were first classified into two subsets based on their cysteinyl-cysteine levels (≤ or >0.045). In the box with high cysteinyl-cysteine (>0.045; right box), there were 10 non-responders and 1 responder. The 19 patients (14 responders and 5 non-responders) with low levels of cysteinyl-cysteine (≤0.045; left box) were further subclassified into two subsets based on the level of threonic acid (≤ or >0.0145). Thirteen of the 14 responders with low levels of cysteinyl-cysteine also showed low levels of threonic acid (≤0.0145; left box). Whereas four of five non-responders among the 19 patients with low levels of cysteinyl-cysteine showed high levels of threonic acid (>0.0145; right box). Approximately ninety percent of patients were then correctly classified as responders or non-responders.

No single metabolite could be used to identify responders and non-responders. However, a decision tree analysis was performed showing that samples can be differentiated based on the levels of cysteinyl-cysteine and threonic acid (Table 1). The patient subset sensitive to PI3K/mTOR inhibitors in vitro was then characterized by low levels of both these metabolites (Figure 3). Cysteinyl-cysteine is a dipeptide composed of two cysteine residues and an incomplete breakdown product of protein catabolism, whereas threonic acid is probably derived from glycated proteins or degradation of ascorbic acid (Human Metabolome Database). This additional and alternative analysis of our metabolomic data further illustrates that our contrasting groups of responder and non-responder AML cells show metabolic differences. 

### 2.4. Modulation of Arachidonic Acid Metabolism Alters PI3K-Akt-mTOR Signaling

In a previous study, we used Western blot to analyze phosphorylation mediators downstream of mTOR in a small group of patients treated with PI3K-mTOR inhibitors [17]. Even though these results have to be interpreted with great care as few patients were studied, the observations suggested that (i) patients differed considerably with respect to the degree of constitutive signaling through the PI3K-Akt-mTOR pathway; and (ii) the heterogeneous antiproliferative effects of PI3K-mTOR inhibitors seen among patients could not be explained by differences in constitutive pathway activation. 

Arachidonic acid metabolism seems to be important for survival and proliferation of various cells, including myeloid cells [21,22]. Arachidonic acid can be metabolized by cyclooxygenase, lipoxygenase, or the cytochrome P450 pathways into a number of metabolites, referred to as eicosanoids. These arachidonic acid derived eicosanoids belong to a complex family of lipid signaling mediators that control many important cellular processes, including cell proliferation, apoptosis, and cell metabolism [21,23]. Therefore, we wanted to investigate whether modulation of the balance between the various pathways of arachidonic acid metabolism would influence PI3K-Akt-mTOR signaling in primary human AML cells. 

In these experiments, we modulated the balance of arachidonic acid metabolism by incubating the cells with indomethacin (a nonselective cyclooxygenase 1/2 inhibitor), and we investigated the effects of this inhibitor on PI3K-Akt-mTOR signaling in primary AML cells derived from five patients showing constitutive signaling throughout this pathway. These five patients showed a wide variation in constitutive pathway activation; this activation was also observed in previous Western blot analyses of the downstream mTOR mediators P70SK6 and p4E-BP1 (see above) [17]. Thus, a variation in the degree of constitutive pathway activation can be detected by both Western blot and phospho-flow, and, therefore, we selected five patient samples with a constitutive, though wide variation of signaling. The variation between these five patients was, in addition, reproduced/documented in independent analysis of cells derived from separate freezing ampullas. 

**Figure 4:**
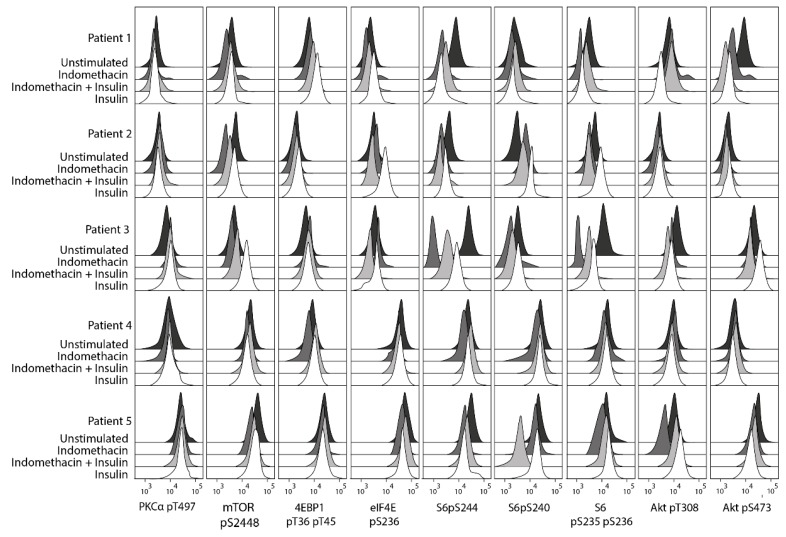
In vitro phospho-signaling analysis of primary AML cells derived from five patients to explore the effects of indomethacin on the PI3K-Akt-mTOR pathway. AML cells were incubated in medium alone, in medium supplemented with 10 μg/mL of either indomethacin or insulin, and in medium supplemented with the combination of insulin and indomethacin. Phosphorylation status of nine mediators were examined. An indomethacin-induced decrease of mTOR pS2448, S6 pS235 pS236, and S6 pS244 was seen for all patients in insulin-free and/or insulin-supplemented cultures, and a decrease of S6 pS240 and Akt pS473 was seen for four of the five patients. The *X*-axis is a log-scale for fluorescence intensity; the *Y*-axis indicates the number of cells.

**Figure 5:**
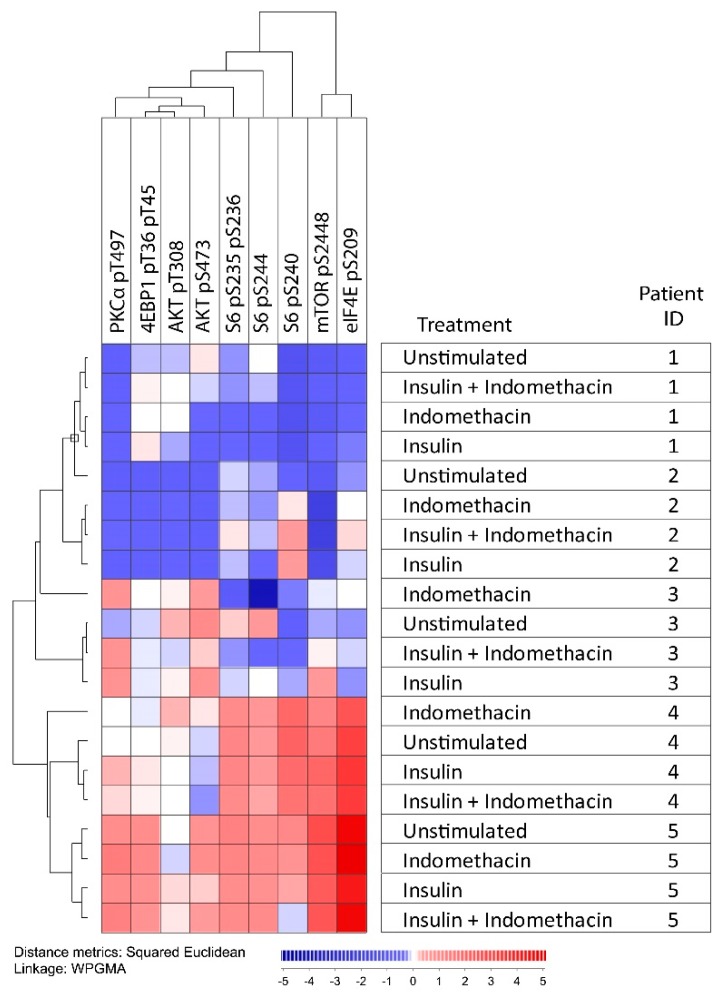
The effect of indomethacin on the activation of PI3K-Akt-mTOR signaling. We investigated the effects of indomethacin on PI3K-Akt-mTOR signaling in primary AML cells derived from five patients. For each sample, we tested AML cells incubated in medium alone, with only indomethacin 10 μg/mL, in medium supplemented with 10 μg/mL insulin, and with the combination of insulin and indomethacin. Phosphorylation status of nine mediators were examined. Red indicates high and blue indicates low phosphorylation/expression of the mediators. All combinations tested for each patient sample also clustered together in the same subclusters for all patients. All values from the flow cytometric analyses were calculated using fold change on the Inverse hyperbolic sine (Arcsinh) scale.

Using a flow cytometry technique, we explored the effects of indomethacin of the PI3K-Akt-mTOR pathway (10 µg/mL, 15-min incubation) for cells incubated in medium alone (i.e., constitutive signaling) and medium supplemented with insulin 10 µg/mL (Figure 4 and Figure 5). Insulin was studied because PI3K-Akt-mTOR is an important pathway downstream of the insulin receptor [24,25], and in vitro studies have shown that insulin is an important growth factor for primary AML cells for a major subset of patients [26]. When performing an unsupervised hierarchical clustering of the overall results, we observed that the four combinations tested (medium alone ± indomethacin, insulin ± indomethacin) for each individual patient sample generally clustered together; showing that differences in pathway signaling between patients were maintained even in the presence of cyclooxygenase inhibition. An indomethacin-induced decrease of mTOR pS2448, S6 pS235 pS236, and S6 pS244 was seen for all patients in insulin-free and/or insulin-supplemented cultures, and for four of the five patients a decrease was seen for Akt pS473 and S6 pS240 (Figure 4). 

Based on our current observations, we conclude that modulation of arachidonic acid metabolism by exposure to indomethacin has only minor effects on the phosphorylation of certain mediators in the PI3K-Akt-mTOR pathway; a similar conclusion can be made also for insulin. Only minor effects were observed on the overall pathway activation profile compared with the observed wide variation in constitutive pathway activation between different patients. Accordingly, our results in Figure 5 illustrate that the wide variation between patients in constitutive pathway activation seems to be maintained also after exposure of the leukemic cells to insulin, as well as after drug-induced modulation of arachidonic acid metabolism, i.e., samples from an individual patient cluster together whereas samples from insulin/indomethacin exposed cells do not cluster together. 

## 3. Discussion

The PI3K-Akt-mTOR signaling network shows constitutive activation in human AML [12]. However, previous experimental studies [17] suggest that the antileukemic effects of pathway inhibitors differ between patients, and the aim of the present study was to further characterize this patient heterogeneity with regard to constitutive PI3K-Akt-mTOR activation/signaling, pathway inhibition, and metabolic regulation. 

For younger patients receiving the most intensive chemotherapy, the overall long-term AML-free survival is only 40–50%. However, the large group of patients above 70–75 years of age are not able to receive this intensive therapy, and are therefore, treated with AML-stabilizing treatment [27]. Many of these elderly patients, as well as younger unfit patients, have an expected survival of only 1–3 months. Thus, new therapeutic strategies are needed both for younger as well as for elderly and unfit patients who can only receive AML-stabilizing treatment; combination therapy including PI3K-Akt-mTOR inhibitors may then be an alternative therapeutic strategy [3,16,18]. However, due to the short survival of many elderly and unfit patients [28,29], they may get only one chance of antileukemic therapy because of the rapid disease progression if this first treatment fails. For this reason, pretreatment identification of patients with high risk of resistant disease will be important. Our present study suggests that metabolic characterization should be further explored as a possible strategy to identify patients with a high risk of resistance to PI3K-Akt-mTOR inhibition, and such patients should then try an alternative strategy as their initial treatment. 

In vitro cultured hierarchically organized AML cell populations show spontaneous apoptosis during the first 4–5 days of culture; for most patients, this is an extensive process [19]. Our previous study showed that PI3K-mTOR inhibitors only have weak influence on this spontaneous or stress-induced in vitro apoptosis, and the variation between patients is limited so that it cannot be used for subset classification [17]. However, a wide variation between patients can be detected when using our ^3^H-thymidine incorporation assay after six days of in vitro culture, i.e., an analysis of the AML cell minority that has been able to survive the initial six days of in vitro culture and still are able to proliferate. Inhibitors of the PI3K-Akt-mTOR pathway were added at the start of the cultures. Our patient classification reflects a combined effect of pathway inhibitors on both survival and proliferation, i.e., the presence of the drug during the initial six days characterized by spontaneous in vitro apoptosis and the ability of the remaining viable cells to still show cytokine-dependent proliferation in the presence of the pathway inhibitors when ^3^H-thymidine incorporation is assayed from day six to seven of in vitro culture. 

We investigated the effect of insulin on phosphorylation of the PI3K-Akt-mTOR-pathway as this pathway is important for insulin signaling [24,25], and insulin is also an important growth factor for in vitro cultured primary human AML cells for a large subset of patients [26]. Our present studies showed that insulin altered the activation/phosphorylation of several mediators; however, the effects were minor and differed between patients. Also, the wide variation in PI3K-Akt-mTOR pathway activation between patients was maintained in the presence of insulin (i.e., the samples exposed to insulin did not cluster together with each other but rather together with the corresponding insulin-free control (Figure 4 and Figure 5)). 

Even though constitutive activation of PI3K-Akt-mTOR signaling in the enriched leukemic cells is seen for most AML patients, resistance to the antiproliferative effect of pathway inhibitors is relatively common. In our present study, we show that resistant patient-derived leukemia cells differ with regard to their metabolomic profile, including the metabolites involved in amino acid and arachidonic acid metabolism. Similar abnormalities are also associated with chemoresistance in other myeloid malignancies [30]. 

Arachidonic acid metabolism is important for survival and proliferation of hematopoietic cells [21,23,31,32,33] and several of its metabolites can influence activation/signaling through the PI3K-Akt-mTOR pathway, e.g., pathway-activating prostaglandins and eicosatetraenoic acid derivatives [31,32,33,34,35]. Our comparison of the metabolite profiles of cell samples representing either responders or non-responders to PI3K-Akt-mTOR inhibition supports the hypothesis that arachidonic acid metabolism is important with regard to susceptibility to these inhibitors. This hypothesis was also supported by our observed effects of modulated arachidonic acid metabolism by indomethacin on mediator phosphorylation, and previous studies in both human chronic myeloid leukemia and in animal models of leukemic stem cells indicating that arachidonic acid metabolism is important for both leukemogenesis and chemosensitivity [20,36].

For other cell types, there are functional links between redox balance, purine metabolism, NADH, proline, and glutamine metabolism, and the citric acid cycle [37,38,39,40,41]. Even though few studies of myeloid cells are available, observations in other cell types suggest links between such metabolic steps and PI3K-Akt-mTOR signaling. Firstly, arachidonic acid metabolites can function as regulators of the PI3K-Akt-mTOR pathway [21,23,42,43], and our present results suggest that this may also be true in human AML. Secondly, there are links between PI3K-Akt-mTOR signaling via free oxygen radicals/redox homeostasis to the NAD/NADH/proline/glutamine/glutamate system [44,45]. Thirdly, proline and glutamine are interconvertible, and glutamine is an important substrate for the energy metabolism in many malignant cells; a link between arachidonic acid and energy metabolism/the citric acid cycle is therefore possible [37,39]. Finally, both arachidonic acid metabolism and PI3K-Akt-mTOR signaling are important for regulation of the peroxisome proliferator activated receptors, a group of transcription factors [21,40]. However, additional studies are needed to clarify the possible contributions of these various steps in human AML. 

Previous experimental studies suggest that altered proline metabolism can be important for the development of cancer chemoresistance, and proline oxidase has been suggested as a possible target in cancer treatment [37,39,41,46]. Our present study suggests that proline/glutamine metabolism may also contribute to resistance of PI3K-mTOR inhibitors in human AML. Our observation of increased levels of eicosatetraenoic acid and docosapentaenoic acid indicates that arachidonic acid may be one of these interacting factors. However, an alternative explanation could be that effects on proline/glutamine only reflect differences in energy metabolisms, and differences in arachidonic acid metabolites may reflect the altered energy/lipid metabolism. This is further supported by previous studies showing that arachidonic acid metabolism is important in murine leukemogenesis and for chemoresistance in human chronic myeloid leukemia [20,36]. The PI3K-Akt-mTOR pathway may represent a link between these two systems through the effect of arachidonic acid metabolites on this pathway and the regulatory effect of mTOR on proline oxidase.

The role of arachidonic acid and its metabolites in normal and malignant hematopoiesis has been reviewed previously [21]. Increased expression of lipoxygenase enzymes has been detected in malignant myeloid cells, and products from this pathway of arachidonic acid metabolism often seem to mediate growth-enhancing and antiapoptotic effects. Our present observations of increased levels of eicosanoids in cells that are resistant to PI3K-Akt-mTOR inhibitors suggest that these metabolites may have such a role in human AML. Furthermore, the effect of arachidonic acid itself seems to differ between cell lines, but proapoptotic effects have been described. Finally, a previous study of primary human AML cells showed that even low levels of indomethacin could reduce the AML cell levels of prostaglandin E_2_, and in their model PGE_2_, could enhance both the spontaneous proliferation as well as Toll like receptor mediated growth enhancement of primary human AML cells [47]. 

## 4. Materials and Methods

### 4.1. AML Patients

The study was approved by the Regional Ethics Committee (REK) (REK III 060.02, 10 June 2002; REK Vest 215.03, 12 March 04; REK III 231.06, 15 March 2007; REK Vest 2013/634, 19 March 2013; REK Vest 2015/1410, 19 June 2015), The Norwegian Data Protection Authority 02/1118-5, 22 October 2002, and The Norwegian Ministry of Health 03/05340 HRA/ASD, 16 February 2004. All AML cell samples were collected after written informed consent. 

The clinical and biological characteristics of those 30 patients included in the metabolic studies are summarized in Table 2. All patients had a high number and/or percentage of peripheral blood blasts; leukemic peripheral blood mononuclear cells could, therefore, be isolated by density gradient separation alone (Lymphoprep, Axis-Shield, Oslo, Norway) and generally contained at least 95% leukemic blasts. The contaminating cells were small lymphocytes. These enriched AML cells were stored in liquid nitrogen until used in the experiments [48]. All the 15 responder patients selected for metabolic profiling had a strong inhibition (i.e., >50% inhibition) of cytokine-dependent AML cell proliferation by both PI3K and mTOR inhibitors, whereas PI3K and mTOR inhibition either increased the proliferation or had a weak antiproliferative effect corresponding to <10% inhibition for the 15 non-responders. 

### 4.2. Drugs

Drugs used in this study included the mTOR inhibitor rapamycin (LC Laboratories, Woburn, MA, USA), the PI3K class I specific inhibitor GDC-0941 (Axon Medchem BV, Groningen, The Netherlands), human insulin (Sigma-Aldrich, St. Louis, MO, USA), and the nonselective cyclooxygenase 1/2 inhibitor indomethacin (Sigma-Aldrich; dissolved in dimethyl sulfoxide (DMSO)). Stock solutions were sterile filtered and stored at −20 °C until used in experiments, thawed only once, and diluted with their respective solvents to obtain the desired final concentrations. 

Indomethacin (Sigma-Aldrich) was tested at a final concentration of 10 µg/mL (corresponding to 28 µM). Previous studies in human as well as murine AML cells often used indomethacin concentrations in the range of 10–50 µM (3.6–18 µg/mL) [49,50,51], and the conventional cyclooxygenase-blocking concentration of indomethacin is considered to be 10–20 µM (for original reference see [50]). However, even indomethacin concentrations as low as 1 µM (0.4 µg/mL) will decrease the in vitro prostaglandin production by primary human acute leukemia cells [47]. Our use of indomethacin 10 µg/mL was based on these previous studies. Finally, in pilot experiments we investigated pharmacological effects after incubation for 7, 10, 15, 30, and 45 min before analyzing the PI3K-Akt-mTOR pathway activation. We decided to incubate cells with the drugs for 15 min because additional effects could not be detected when using longer incubations. 

### 4.3. Analysis of PI3K-Akt-mTOR Activation

Flow cytometry was used to examine the basal expression of 18 mediators in the PI3K-Akt-mTOR pathway/network in the AML cells. Cryopreserved and thawed primary leukemic cells were incubated for 20 min in RPMI-1640 (Sigma-Aldrich) before being directly fixed in 1.5% paraformaldehyde (PFA) and permeabilized with 100% methanol. The cells were subsequently rehydrated by adding 2 mL phosphate-buffered saline (PBS), gently re-suspended, and then centrifuged. The cell pellet was washed twice with 2 mL PBS and resuspended in 150 μL PBS supplemented with 0.1% bovine serum albumin (BSA) (Sigma-Aldrich). Washed cells were blocked with immunoglobulin (Octagam; Octapharma, Jessheim, Norway) and 1% BSA, and then split evenly into nineteen new tubes (1 × 10^5^ cells per sample) before staining. All staining panels included the same live/dead discriminator, either FITC or Alexa Fluor^®^ 647 Mouse anti-Cleaved PARP (Asp214); an unstained sample was also included. Three directly conjugated dyes were used: (i) Alexa Fluor^®^ 647 was used for PTEN, PDPK1 pS241, PKCα, PKCα pT497, Akt pS473, 4EBP1 pT36 pT45, elF4E pS209, S6 pS244, and mTOR; (ii) phycoerythrin (PE) for Akt total, Akt pT308, mTOR pS2448, and S6 pS240; and (iii) V450 for S6 pS235 pS236. Antibodies were purchased from BD Pharmingen (Franklin Lakes, NJ, USA), except for anti-mTOR that was purchased from Cell Signal Technology (Danvers, MA, USA). Four of the antibodies were unconjugated (anti Raptor, Tuberin, FKBP38, and RHEB; all from Abcam; Cambridge, UK) and required secondary antibody-conjugated Alexa Fluor^®^ 647 (BD Pharmingen). Together these mediators represent the main steps in the PI3K-Akt-mTOR pathway and they were selected to provide an extended phosphorylation profile of this pathway. Finally, in our pharmacological studies, AML cells were incubated with human insulin 10 µg/mL (Sigma-Aldrich) and/or indomethacin 10 µg/mL (Sigma-Aldrich; dissolved in DMSO); final concentration in the medium 0.5%) or a DMSO control solution for 15 min before flow cytometric analysis as described above. Flow cytometry analysis was acquired on a BD FACS Verse 8-color flow cytometer (BD Biosciences, San Jose, CA, USA) and data analysis performed using FlowJo 10.0.7 software (Tree Star, Inc., Ashland, OR, USA). 

### 4.4. Analysis of Cytokine-Dependent Proliferation in Presence of PI3K-mTOR Inhibitors

As described in detail previously [52,53]; AML cells (5 × 10^4^ cells/well) were cultured in flat-bottomed microtiter plates (150 µL/well) in Stem Span SFEM^TM^ serum-free medium (Stem Cell Technologies; Vancouver, BC, Canada) alone or in medium supplemented with granulocyte-macrophage colony-stimulation factor (GM-CSF), stem cell factor (SCF) and Fms like tyrosine kinase 3 ligand (Flt3L) [54]. All cytokines were purchased from Peprotech (Rocky Hill, NJ, USA) and used at 20 ng/mL. All drugs were added on the first day of culture, whereas 37 kBq/well of ^3^H-thymidine (Perkin Elmer; Waltham, MA, USA) was added after 6 days, and nuclear incorporation was assayed after seven days of culture. The mTOR inhibitor rapamycin and the PI3K inhibitor GDC-0941 were added at a final concentration of 100 nM on the first day of culture [55]. 

### 4.5. Metabolomic Analysis

The metabolomic analyses and sample preparations were performed by Metabolomic Discoveries GmbH (Potsdam, Germany) [56]. Briefly, non-targeted metabolite profiling of cells included analyses by gas chromatography/mass spectrometry (GC-MS) and Liquid Chromatography Quadrupole-Time of Flight (LC-QTOF)/MS; metabolites could then be analyzed in the range of 50–1700 Da with an accuracy up to 1–2 ppm and a resolution of mass/Δmass = 40,000. Metabolites measured in the LC were annotated according to their accurate mass and subsequent sum formula prediction. Metabolite profiles were explored by the platforms of Metabolomic Discoveries and Small Molecule Pathway Database (BioVariance, Munich, Germany). The lists of the 627 detected and the 128 annotated metabolites are presented in the Supplementary Materials presenting the identity/accurate mass@retention time, the *p*-value, and responder/non-responder ratio.

### 4.6. Bioinformatical and Statistical Analyses

Bioinformatic analyses were performed using the J-Express 2012 software (MolMine AS, Bergen, Norway). For hierarchical clustering analysis, all values from cytometric analyses were calculated using fold change on the Inverse hyperbolic sine (Arcsinh) scale. The median signal for each phospho-protein was used as reference value for the calculation of basal phosphorylation. Complete linkage and Squared Euclidean correlation were used as linkage method and distance measurement, respectively. Statistical analyses were performed using the IBM Statistical Package for the Social Sciences (SPSS) version 23 (Chicago, IL, USA), and *p*-values < 0.05 were regarded as statistically significant. 

## 5. Conclusions

Despite the metabolic heterogeneity of the non-responders to PI3K-Akt-mTOR inhibitors, there are distinct metabolic differences between responders and non-responders. Our present results are consistent with the hypothesis that differences in arachidonic acid metabolism together with differences in proline and/or energy metabolism are associated with differences in susceptibility to pathway inhibitors. The possible importance of such differences should be considered when planning or analyzing future clinical studies with PI3K-Akt-mTOR inhibitors and when designing combination therapy for various AML patient subsets [57].

## Figures and Tables

**Table 1:** A description of annotated metabolites that differed significantly between the two patient groups and were sensitive (responders) or insensitive (non-responders) to the in vitro antiproliferative effect of phosphatidylinositol-3-kinase-Akt-mechanistic/mammalian target of rapamycin (PI3K-Akt-mTOR) inhibition.

Metabolite	*p*-Value	Ratio * Responder versus Non-Responder	Short Description
↓Allose	0.037	−0.875	Sugar metabolism. Possibly involved in cell cycle regulation.
↓Citric acid	0.005	−1.262	Energy metabolism, citric acid cycle.
↓Cysteinyl-cysteine	0.006	−1.471	Dipeptide
↓Glutamine	0.029	−0.737	Non-essential amino acid, important for nucleic acid synthesis. Energy metabolism, conditionally essential during catabolic states.
↓Indoleacrylic acid	0.047	−0.426	Involved in tryptophan metabolism.
↓Isocitric acid	0.029	−0.698	Substrate of the citric acid cycle.
↑Phosphatidyl inositol (18:0/0:0)	0.040	0.765	Lipid metabolism, cell membrane constituents.
↑Phosphatidyl inositol (15:1(9Z)/22:6(4Z,7Z,10Z,13Z16Z19Z))	0.025	0.809	Lipid metabolism, cell membrane constituents.
↓Phosphonic acid (8:0/8:0)	0.009	−1.660	Lipid metabolism
↓Proline	0.046	−0.611	Non-essential amino acid, synthesized from glutamic acid and also other amino acids, energy metabolism.
↓Taurine	0.035	−1.0524	Sulfur amino acid not incorporated into protein; adults can synthesize taurine from cysteine. Stabilizes cell membranes, regulates ion transport.
↓2-amino-4-hydroxy-propiophenone	0.021	−0.744	Lipid metabolism
↓4-phenyl-1,2,3-thiadiazole	0.041	−1.024	Inhibitor of cytochrome P450 enzymes that regulate arachidonic acid metabolism.
↓4,7,10,13-eicosatetraenoic acid	0.021	−0.983	Arachidonic acid metabolite, possibly influencing the leukotriene B4 (LTB4) pathway; expression of the LTB4 receptor (BLT1) may be altered in myeloid leukemia cells.
↓4,7,10,13,16-docosapentaenoic acid	0.042	−0.766	Fatty acid and arachidonic acid metabolism, an intermediate between eicosapentaenoic acid and docosahexaenoic acid, precursor of prostanoids that are only formed from docosapentaenoic acid.

* Responders versus non-responders were compared as the log_2_-ratio. The arrows to the left in the table indicate whether the mean metabolite levels were decreased (↓) or increased (↑) in responder cells relative to the non-responder cells. The information in this table is based on PubChem and Human Metabolome databases.

**Table 2:** Important clinical and biological characteristics of responders and non-responders to of phosphatidylinositol-3-kinase- mechanistic/mammalian target of rapamycin (PI3K-mTOR) inhibitors.

ID	Gender	Age	Previous Hematological Malignancy or Chemotherapy	FAB	CD34	Karyotype		*Flt3* Mutation	*NPM-1* Mutation
						Abnormality	Classification		
**Responders**									
1	F	45	Chemotherapy	M4	Negative	Normal	Normal	wt	ins
2	F	63		M4	Positive	Normal	Normal	ITD	wt
3	M	72		M5	Negative	Normal	Normal	wt	ins
4	M	29	Relapse	M4	Positive	Normal	Normal	ITD	ins
5	F	80		M2	Positive	Complex	Adverse	wt	wt
6	F	36		M4	Positive	Normal	Normal	wt	nt
7	F	75		M1	Positive	nt		ITD	wt
8	M	71	Relapse	M2	Negative	Normal	Normal	G835	
9	M	35		M2	Positive	Normal	Normal	wt	wt
10	M	72	Myelodysplastic syndrome	M1	Positive	Complex	Adverse	wt	
11	F	64	Chemotherapy	M2	Negative	Normal	Normal	ITD	ins
12	F	59	Chemotherapy	M5	Negative	Normal	Normal	ITD	ins
13	M	58		M5	Positive	Normal	Normal	wt	wt
14	F	59	Chemotherapy	M4	Negative	Normal	Normal	ITD	ins
15	F	75		M4	Positive	Normal	Normal	ITD	wt
**Non-responders**									
16	F	29	Chemotherapy	M5	Positive	Normal	Normal	ITD+Asp835	wt
17	M	24		M2	Positive	Multiple	Adverse	nt	wt
18	F	82		M4	Positive	Normal	Normal	ITD	wt
19	F	77		M1	Negative	nt		nt	ins
20	M	84		M1	Positive	Multiple	Adverse	wt	wt
21	M	53		M0	Positive	13	Intermediate	wt	wt
22	M	65		M5	Negative	Normal	Normal	ITD	ins
23	F	46		M1	Positive	inv(16)	Favorable	wt	wt
24	F	70		M4	Negative	nt		wt	ins
25	M	33	Chemotherapy	M1	Positive	Normal	Normal	wt	wt
26	F	77		M1	Positive	nt		nt	wt
27	M	76		M0	Positive	Normal	Normal	wt	wt
28	M	60		M4	Positive	Normal	Normal	ITD	wt
29	M	36		M5	Positive	+8, +22, inv(16)	Favorable	ITD	wt
30	F	67		M5	Negative	t(9,11), +19	Intermediate	wt	wt

The table shows the gender (M, male; F, female) and age (years) of the individual patients at diagnosis. The FAB classification was used to classify morphological and/or histochemical signs of differentiation. Cytogenetic abnormalities were classified according to the medical research council (MRC) criteria. The detection of Fms like tyrosine kinase 3 (Flt3) (ITD, internal tandem duplications) or nucleophosmin (NPM)-1 insertions (ins) is also indicated in the table. Complex karyotype means at least three abnormalities [1]. FAB: The French-American-British (FAB) classification system; nt: not tested; wt: wild type.

## Supplementary Materials

Supplementary materials can be found at http://www.mdpi.com/1422-0067/19/2/382/s1.

## Abbreviations

4EBP1	Translation initiation factor 4E-binding protein 1
AML	Acute myeloid leukemia
DMSO	Dimethyl sulfoxide
DNA-PK	DNA-dependent protein kinase
FAB	The French-American-British () classification system
Flt3	Fms like tyrosine kinase 3
Flt3L	Flt3 ligand
GC-MS	Gas chromatography-mass spectrometry
GM-CSF	Granulocyte-macrophage colony-stimulation factor
ins	Insertions
ITD	Internal tandem duplications
LC-QTOF/MS	Liquid Chromatography Quadrupole-Time of Flight MS
mRNA	Messenger RNA
mTOR	Mechanistic/mammalian target of rapamycin
mTORC	mTOR complex
NPM	Nucleophosmin
PBS	Phosphate-buffered saline
PCA	Principal component analysis
PDK1	3’phosphoinositide-dependent kinase 1
PFA	Paraformaldehyde
PI3K	Phosphatidylinositol-3-kinase
PIP2	Phosphatidylinositol (4,5)-bisphosphate
PIP3	Phosphatidylinositol (3,4,5)-trisphosphate
PRAS40	Proline-rich Akt-substrate-40
RHEB	Ras homolog enriched in brain
S6PK	S6 ribosomal protein kinase
SCF	Stem cell factor
SPSS	Statistical Package for the Social Sciences
TSC	Tuberous sclerosis complex
elF4E pS209	eukaryotic translation Initiation Factor 4E
PKCα	Protein kinase C α
PTEN	Phosphatase and tensin homolog
